# DNA Methyltransferases in Cancer: Biology, Paradox, Aberrations, and Targeted Therapy

**DOI:** 10.3390/cancers12082123

**Published:** 2020-07-31

**Authors:** Jiayu Zhang, Cheng Yang, Chunfu Wu, Wei Cui, Lihui Wang

**Affiliations:** 1 Department of Pharmacology, Shenyang Pharmaceutical University, Shenyang 110016, China; 18745774959@163.com (J.Z.); 18275366347@163.com (C.Y.); wlhcw@163.com (C.W.); 2 Benxi Institute of Pharmaceutical Research, Shenyang Pharmaceutical University, Benxi 117004, China

**Keywords:** DNA methyltransferases, cancer, DNMT aberrations, therapy

## Abstract

DNA methyltransferases are an essential class of modifiers in epigenetics. In mammals, DNMT1, DNMT3A and DNMT3B participate in DNA methylation to regulate normal biological functions, such as embryo development, cell differentiation and gene transcription. Aberrant functions of DNMTs are frequently associated with tumorigenesis. DNMT aberrations usually affect tumor-related factors, such as hypermethylated suppressor genes and genomic instability, which increase the malignancy of tumors, worsen the prognosis for patients, and greatly increase the difficulty of cancer therapy. However, the impact of DNMTs on tumors is still controversial, and therapeutic approaches targeting DNMTs are still under exploration. Here, we summarize the biological functions and paradoxes associated with DNMTs and we discuss some emerging strategies for targeting DNMTs in tumors, which may provide novel ideas for cancer therapy.

## 1. Introduction

Regulation of gene expression is an area of intense research interest. In particular, great attention has been paid to the epigenetic phenomenon where heritable changes in gene expression occur with no alteration of the DNA sequence [1]. The discipline of epigenetics is defined as the study of molecular processes that affect information flow between the same DNA sequence and variable gene expression patterns. At present, epigenetics spans many research fields, such as oncology and immunology [2]. Aberrant epigenetic processes can lead to changes in gene function and transformation of malignant cells. Global alterations in epigenetic modifications are a hallmark of cancer. Cancer has traditionally been regarded as a hereditary disease, and is now recognized as resulting from epigenetic abnormalities related to genetic changes. In the rapidly developing field of cancer epigenetics, the relevant epigenetic modifications mainly include DNA methylation, histone modification and RNA regulation [3].

DNA methylation is one of the earliest discovered and most deeply studied epigenetic regulatory mechanisms [4]. It is a major form of epigenetic modification of genomic DNA and an important means of regulating genomic function. The methylation status of the genome is mainly the result of joint regulation by DNA methyltransferases (DNMTs) and DNA demethylases [5]. Using S-adenosylmethionine (SAM) as the methyl donor, DNMTs add a methyl group (CH3) to the C5 position of the cytosine ring in a cytosine-phosphate-guanine (CpG) dinucleotide pair to yield 5′-methyl-cytosine [6]. DNMTs play a crucial role in many biological processes, such as embryo development, cell differentiation and gene transcription [7]. In mammalian genomic DNA, 3% to 5% of cytosine exists in the form of 5-methylcytosine, and 70% of 5-methylcytosine is located within CpG sequences. Non-methylated CpG sequences are associated with housekeeping genes and tissue-specifically expressed genes, which suggests that CpG methylation plays an important role in gene expression [8]. Genes are located on chromosomes in the context of chromatin, so histone modifications are also indispensable in the process of gene regulation. Histone modification refers to the methylation and acetylation of histone proteins under the action of related enzymes such as histone methyltransferases, histone acetyltransferases, histone deacetylases, etc. Histone methylation mainly occurs on lysine and arginine residues of histones H3 and H4, histone methyltransferase can selectively methylate lysines and arginines in the tails of histones, and synergistically induce transcriptional activity. Moreover, when histone acetylation in chromatin is in a loose state, which is conducive to gene expression [9,10]. In addition to the coding part of nucleic acid plays an important role in gene expression, and functional non-coding RNA also plays an important role in gene expression. There are long and small non-coding RNAs. Long non-coding RNA plays a cis-regulatory role at the gene and even the entire chromosome level, and small non-coding RNA regulates gene expression at the genomic level. Small non-coding RNA can mediate the degradation of mRNA, induce changes of chromatin structure, and determine how cells differentiate. Small non-coding RNA can also degrade foreign nucleic acid sequences to protect the cell’s genome [11,12].

This review focuses on induction of DNA methylation by DNMTs and specifically introduces this process in the context of tumor research [3]. A large number of studies have shown that aberrant DNMTs can cause tumorigenesis [13,14]. Therefore, we describe the phenomena associated with DNMT alterations, discuss current therapies that target DNMTs, and provide more ideas for targeting DNMTs in cancer.

## 2. DNMTs

**Figure 1:**
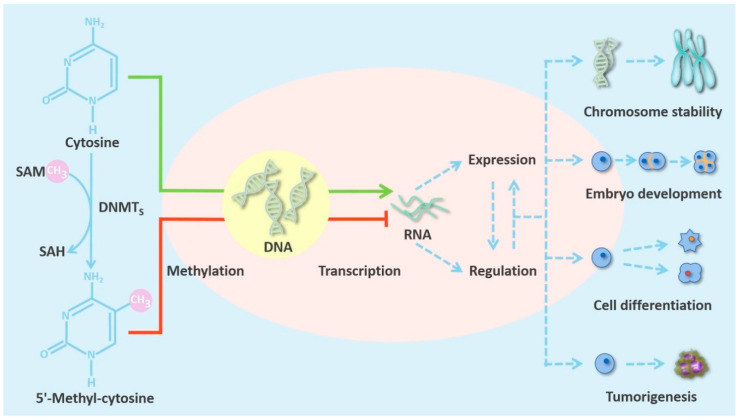
The characteristics of DNMTs in mammalian cells. DNMTs catalyze DNA methylation, which alters patterns of gene regulation and expression. This in turn affects chromosome stability, embryo development and cell differentiation. Aberrant DNMTs can induce tumorigenesis.

DNMTs are an extremely important class of epigenetic regulatory enzymes. They catalyze DNA methylation, a normal endogenous modification of eukaryotic DNA, which is vital for life [15]. This process, which is illustrated in Figure 1, can cause alterations in gene expression, but does not change the sequence of the DNA or of the gene product [16].

### 2.1. Family Members

The first eukaryotic DNMT was successfully cloned in 1988 [17]. The DNMT family has five members, which are DNMT1, DNMT2, DNMT3A, DNMT3B, and DNMT3L. The main function of DNMT1 is to maintain the methylation status after DNA synthesis and DNMT2 is an RNA methyltransferase, which methylates multiple tRNAs [18]. DNMT3A and DNMT3B are involved in the de novo methylation of DNA [19], while DNMT3L is a DNMT3-like protein that lacks a catalytic domain. DNMT3L can interact with DNMT3A and DNMT3B to improve their catalytic activity and positively regulate DNA de novo methylation [20]. DNMT3A and DNMT3B catalyze the methylation of different genomic DNA regions in vivo. DNMT3A has higher methylation activity on naked DNA and the naked part of nucleosomal DNA than DNMT3B. DNMT3A hardly methylates DNA in the core region of nucleosomes, while DNMT3B significantly methylates this DNA, although the activity is low. Studies have shown that the preferential methylation activity of DNMT3A on naked nucleosomal DNA and the significant methylation activity of DNMT3B on the DNA in the nucleosome core contribute to their significant methylation of genomic DNA in vivo. The expression of DNMTs at different times during embryonic development may be one of the reasons why these enzymes methylate different DNA regions [21].

In mammals, DNA methylation is mainly mediated by three proteins, DNMT1, DNMT3A, and DNMT3B, which are localized in the nucleus [22]. This review will focus on these three main enzymes.

### 2.2. Locations and Domains

The human *DNMT1* gene, which is located on chromosome 19, has a total protein coding region of 4851 bp and encodes a protein of about 183 kDa. It is generally believed that the DNMT1 protein consists of three domains, namely the N-terminal regulatory domain, the C-terminal catalytic domain, and a central junction region [23]. The N-terminal regulatory domain is responsible for localizing the protein to the nucleus. Most of the C-terminal catalytic domain is evolutionarily conserved, with binding sites for various substrates such as cytosine. The central junction region, which connects the other two domains, contains repeated glycine-lysine dipeptide sequences [17].

The human *DNMT3A* gene, located on chromosome 2, has a total protein coding region of 2172 bp and encodes a protein of about 130 kDa, while the *DNMT3B* gene, on chromosome 20, has a total protein coding region of 2538 bp and encodes a protein of about 95 kDa [24]. DNMT3A and DNMT3B have similar structures, consisting of three domains: the N-terminal proline-tryptophan-tryptophan-proline (PWWP) domain [25], the cysteine-enriched ATRX-DNMT3-DNMT3L (ADD) zinc-binding region and the C-terminal methyltransferase catalytic region. The PWWP domain mediates the specific binding of DNA. The cysteine-enriched zinc-binding region interacts with unmodified histones to enhance methyltransferase activity. The C-terminal catalytic region maintains the stable function of the enzyme and catalyzes DNA methylation [26].

The N-terminal regulatory domain of DNMT1, DNMT3A and DNMT3B comprises a proliferating cell nuclear antigen binding domain (PBD), a nuclear localization signal (NLS), a cytosine-rich zinc finger DNA binding motif (ATRX), a polybromo homology domain (PHD), and a PWWP tetrapeptide chromatin binding domain. The C-terminal catalytic domain contains 10 different characteristic motifs, of which 6 motifs (I, IV, VI, VIII, IX and X) are evolutionarily conserved in mammals. The methylation reaction is initiated by conserved cysteine residues in motif IV, which mediate nucleophilic attack on the cytosine ring [27].

### 2.3. Normal Biological Characteristics

In general, DNA methyltransferases are involved in important biological activities such as preserving chromosome stability and genome integrity, embryo development, cell differentiation, and growth of organisms [7]. DNMT1, which maintains methylation, is believed to have a high affinity for hemimethylated DNA templates. DNMT3A and DNMT3B are considered to be the main de novo methyltransferases that change the methylation status of unmethylated CpG sites [28]. De novo methyltransferases are mainly active in the development of embryonic stem cells, where they identify unmethylated cytosines in DNA and establish a new methylation pattern. Moreover, DNMT3B is highly expressed in totipotent embryonic stem cells in the early developmental stage, when DNA methylation occurs. After that, DNMT3A is widely expressed in mesenchymal cells, and the expression level of DNMT3B is one order of magnitude higher than that of the widely expressed DNMT3A [21]. Both DNMT3A and DNMT3B are downregulated in most differentiated somatic cells [29]. The maintenance methyltransferase DNMT1 is associated with early embryo implantation in the endometrium, where it is an important component of the DNA replication complex. After identifying the methylation site, it catalyzes the methylation of DNA hemimethylation sites through nucleophilic attack to maintain the established methylation pattern by de novo methyltransferases [30]. Table 1 summarizes information from the Uniprot portal (www.uniprot.org) to show the expression levels of DNMTs in normal tissues.

The DNMT-mediated methylation of CpG sites can make chromatin structure highly spiral, prevent the activation of promoters, and facilitate interaction with methylated CpG-binding proteins such as MeCP2, which recruits histone deacetylase [44]. These changes can inhibit gene expression. Methylated DNA can also directly inhibit the binding of methylation-sensitive transcription factors, such as E2F, CREB, etc., so that transcription is blocked [45,46]. At the same time, histones can also affect DNA methylation. The PWWP motif in DNMT3A recognizes the modification H3K36me3 (trimethlyation of lysine 36 in histone H3). The interactions between the PWWP domain and the H3K36me3 modification can increase the ability of DNMT3A to methylate nucleosome DNA [47]. Studies have shown that DNMT3B, not DNMT3A, is responsible for methylating the bodies of transcribed genes, and this function is also realized by PWWP recognizing the H3K36me modification [48]. DNA methylation has been proved to overlap with H3K27me3 at multiple sites throughout the genome, and unmethylated CpG sites play a role in guiding polycomb repressive complex 2 (PRC2) to bind to its target genes [49].

DNMTs are also closely linked with the function of the nervous system. Many studies have found that DNA methylation is related to transcriptional silencing. For example, memory formation during fear conditioning involves methylation and silencing of the memory suppressor gene *PP1*. The inhibition of DNMTs prevents the increase of *PP1* methylation, resulting in abnormal gene transcription during the memory consolidation period [50]. DNMT activity is also involved in the process of cellular aging, and there is a widespread decline in DNA methylation in many aging tissues [51].

### 2.4. Pathological Characteristics

Genetic mutations caused by chemical carcinogens or pathological conditions, may directly affect the functions or levels of DNMTs, which may in turn cause the genome-wide methylation profile alterations and cancer-promoting changes in gene expression, such as increasing genomic instability and the expression of oncogenes and decreasing the expression of tumor suppressor genes [52,53,54].

Recent studies have found that gene expression abnormalities caused by alterations in DNMT activity and function are closely connected with the occurrence and development of various cancers [55,56]. In mammals, some CpG dinucleotides are dispersed in the genome, while others occur in dense clusters called CpG islands. Dispersed CpGs are hypermethylated; in contrast, CpG islands are usually unmethylated and are often located in gene promoter regions that regulate transcriptional activation and repression. In normal cells, CpG islands in the promoters of tumor suppressor genes (TSGs) are unmethylated, and in these regions chromatin is in an open conformation, so gene transcription can proceed normally [57]. However, genome-wide hypomethylation and promoter hypermethylation are hallmarks of many cancers and are associated with tumorigenesis. Genome-wide hypomethylation can lead to increased genomic instability [58,59]. More importantly, hypermethylation of the CpG islands in gene promoters can silence TSGs and affect key cellular processes such as the cell cycle, apoptosis, DNA repair, cell interactions, and angiogenesis [60]. Tumor therapy focuses on restoring transcription of TSGs which is inhibited by CpG island methylation. Therefore, it is particularly important to explore aberrations in DNMT activity. The essential processes described above are shown in Figure 1 and Table 1.

Furthermore, in addition to investigating DNMT alterations at the cellular level, the researchers also established DNMT animal models so as to study DNMT pathological characteristics in vivo and in vitro. Belinsky et al. utilized a mouse model containing an allele with the *DNMT1* gene disrupted to study the functional role of DNMT in tumor formation [61]. The reduction of DNMT1 led to a decrease in the incidence of lung cancer induced by tobacco carcinogens, and DNMT activity was also reduced in pneumocytes that could give rise to lung cancer. Moreover, it has been reported that DNMT3A may directly inhibit the upregulation of DNMT3B in lymphoma in animal models. Inactivation of the *DNMT3B* gene accelerated the development of lymphoma in *DNMT3A*-deficient mice, which indicates that the upregulation of DNMT3B inhibits disease progression in DNMT3A-deficient lymphoma [62]. In mice treated with skin cancer-inducing chemicals, deletion of *DNMT3A* increases the number of carcinogen-induced squamous cell carcinomas without affecting tumor progression. When *DNMT3A* and *DNMT3B* are both deleted, the squamous cell carcinomas become more invasive and metastatic [63]. These studies show that loss of DNMTs has a major impact on organisms.

### 2.5. The Paradox of DNMTs

An interesting phenomenon was found when the effects of aberrant DNMTs were characterized in tumors. Some studies showed that overexpression of the DNMT3A protein in vulvar squamous cell carcinomas and pituitary adenomas is associated with malignant characteristics such as high invasiveness and recurrence [31,37]. Similarly, increased expression of DNMT3A in lung cancer leads to the proliferation and metastasis of tumor cells. Yan et al. showed that microRNA-101 (miR-101) expressed significantly inhibited DNMT3A activity corresponding to miR-101 binding site. While in the presence of miR-101, the expression of DNMT1 and DNMT3B did not change significantly, indicating that DNMT3A is a direct target of the miR-101 in lung cancer cells. Therefore, expression of miR-101 will reduce the expression of endogenous DNMT3A, which will decrease the overall DNA methylation. Cadherin-1 (CDH1) is a known inhibitor of lung metastasis. The methylation status of CpG sites in the *CDH1* promoter was analyzed in miR-101-transfected lung cancer cells by bisulfite analysis. The proportion of unmethylated sites was much higher in the miR-101-transfected cells than in control cells, which indicates that CDH1 is re-expressed via promoter hypomethylation [64]. Similarly, miR-708-5p can directly inhibit the translation of DNMT3A, resulting in a significant reduction in promoter DNA methylation levels and upregulation of the tumor suppressor gene CDH1. Upregulation of CDH1 reduces the activity of Wnt/β-catenin signaling in non-small cell lung cancer cells, thereby impairing the stem cell characteristics of NSCLC cells [65].

In complete contrast, Husni et al. pointed out that the lack of DNMT3A facilitate tumor progression [66]. Gao et al. used a mouse lung cancer model to verify that DNMT3A deficiency can promote tumor growth and progression and detected no significant difference in the abundance of DNMT1 or DNMT3B mRNAs in DNMT3A-deficient and WT tumors [67]. Raddatz et al. proved that DNMT3A is necessary to effectively maintain the methylation of active chromosome domains and DNMT3A-deficient tumors showed moderate gene deregulation in these domains. Through overall methylation analysis, it was found that the proportion of fully methylated CpGs in DNMT3A knockout tumors decreased significantly, while the proportion of partially methylated CpGs increased accordingly. This study revealed the conserved features of genomic methylation in cancer cells, DNMT3A deficiency increased the instability of the whole genome, confirming the role of DNMT3A in maintaining the methylation pattern of cancer DNA [68].

**Figure 2:**
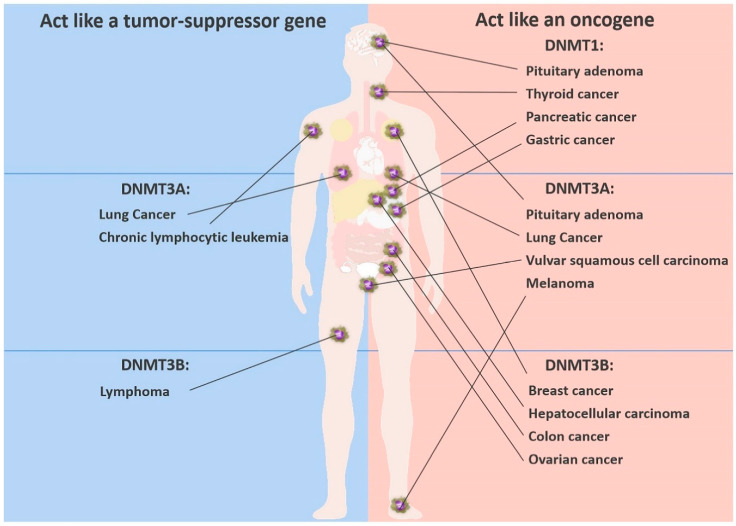
The DNMT paradox and the different roles of DNMTs in various cancers in mammals. DNMT3A can act both as an oncogene and as a tumor suppressor gene in lung cancer. Other DNMTs play different roles in other cancers.

These two completely opposite findings suggest that *DNMT3A* can act both as an oncogene and as a tumor suppressor gene in lung cancer (Figure 2). At present, it is less clear whether the paradox applies to the other DNMTs in other cancers. The paradoxical effect of DNMT3A led us to ask the following questions: How do the alterations in DNMTs affect the tumor? And what strategies can be used to target DNMTs in tumors?

In the next sections, we systematically introduce the role of DNMTs in different tumors as well as the treatment approaches. We believe that the impact of DNMT alterations on cancers needs to be further investigated to provide potential routes to cancer therapy. Moreover, understanding the molecular roles of aberrant DNMTs in tumorigenesis will lay the theoretical foundations for clinical research and development of new drugs.

## 3. DNMT Aberrations in Cancer

**Figure 3:**
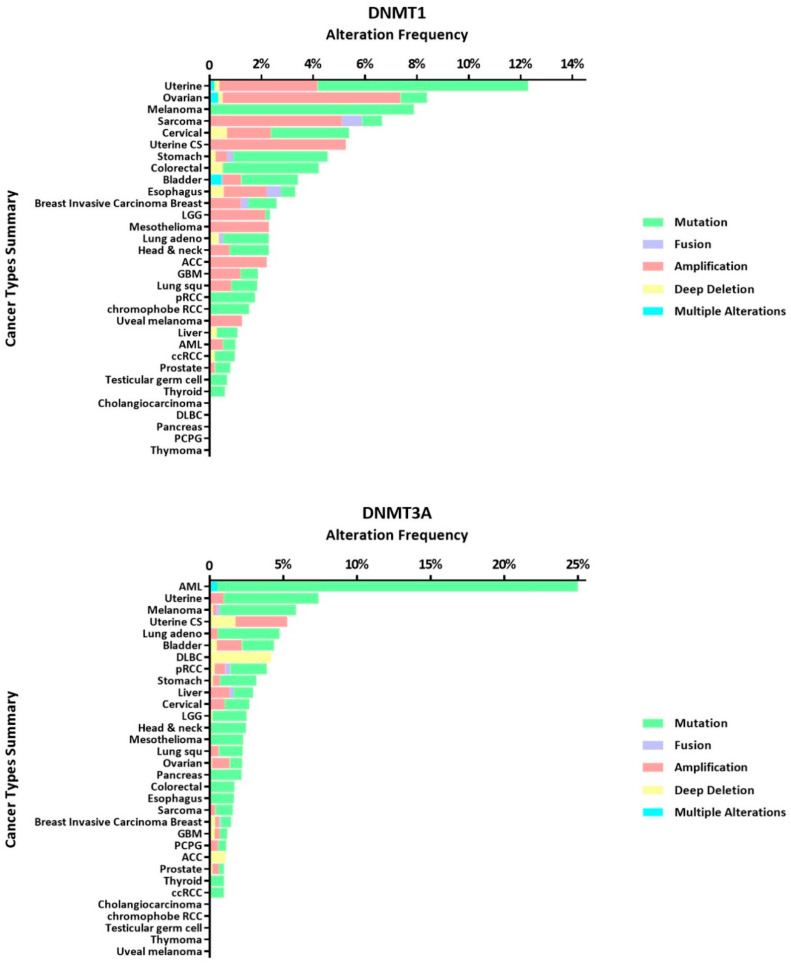

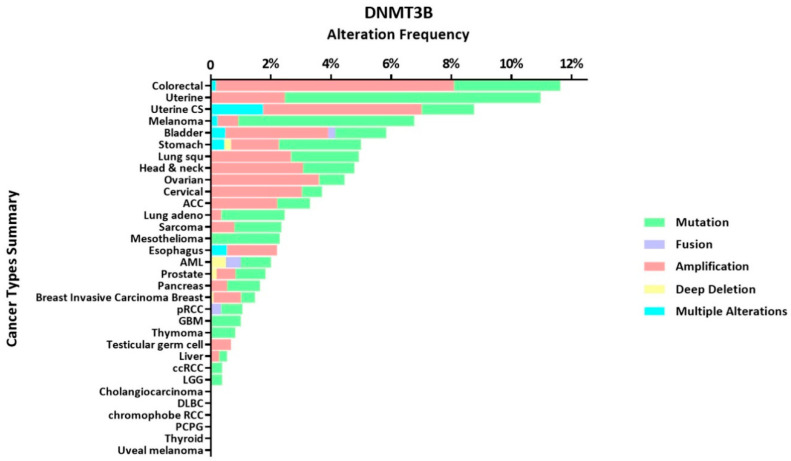
DNMT alteration frequency in cancer types summary. DNMT1, DNMT3A, DNMT3B occur mutation, fusion, amplification, deep deletion and multiple alterations in variety cancer types from cBioPortal datasets.

Carcinogenesis of normal cells is often related to changes in the genetic material [69,70]. DNMT alterations have been frequently observed by many researchers in various types of tumors, which indicates that they often accompany the occurrence and development of tumors. We utilize publicly available datasets (TCGA PanCancer Atlas Studies) from cBioPortal for Cancer Genomics (www.cbioportal.org) to better present DNMT alternation frequency in variety cancer types (see Figure 3). Therefore, analysis of DNMT abnormalities in tumors may uncover a way to restore DNA stability, which may lead to new methods to treat tumors. The aberrations mentioned here mainly refer to mutations, high expression and low expression.

### 3.1. DNMT1 Aberrations

Mutations in *DNMT1* generally cause neurological diseases [71]. In addition, Kanai et al. found that the coding exons of the *DNMT1* gene were mutated in 7% of human colorectal cancers tested, which was the first evidence that *DNMT1* is mutated in human cancer [32].

High expression of DNMT1 has been observed in a variety of tumors and is a characteristic change of tumor cells. Li et al. analyzed 20 pancreatic cancer cell lines and found that 16 of them had DNMT1 expression more than twice that of normal cells [72]. Etoh et al. found that DNMT1 could not be detected in normal epithelium, but was detected in most gastric cancers. Furthermore, the overexpression of DNMT1 protein in gastric cancer tissues is significantly related to decreased E-cadherin expression, which indicates that an increase in DNMT1 expression will enhance the migration ability of the cancer cells. Researchers have investigated the relationship between the high expression of DNMT1 protein and the DNA methylation status of multiple CpG islands (*p16*, *hMLH1*, *E-cadherin* and *THBS-1*) [34]. For patients who show DNA methylation of a CpG island in both non-cancerous mucosa and cancer, the signal intensity of the product of methylated DNA is increased in the cancer cells compared with the corresponding non-cancerous mucosa.

Belinsky’s team discovered that in lung cancer, in addition to affecting the methylation status of cytosine, DNMTs can also bind to histone deacetylase (HDAC) and mediate the formation of heterochromatin around abnormally methylated promoters [61]. They also found that a 2-fold overexpression of the *DNMT1* gene increased DNA methylation and tumorigenic transformation in NIH3T3 cells. Similarly, the work of Lin et al. indicates that overexpression of DNMT1 causes abnormal regulation of the p53/Sp1 pathway, resulting in hypermethylation of multiple TSGs (*p16*, *RARβ*, *FHIT*, *RASSF1A* and *hRAB37*), and eventually leading to the occurrence of lung cancer with poor prognosis [35].

### 3.2. DNMT3A Aberrations

Among *DNMT1*, *DNMT3A* and *DNMT3B*, mutations in *DNMT3A* have been reported most frequently in cancer. *DNMT3A* mutation is one of the earliest that occurs during the development of hematopoietic malignancies. Most researchers believe that DNMT3A mutation indicates a poor prognosis in patients with AML. The R882 site of *DNMT3A* is the most likely to mutate. The most common mutation is R882H, and the others are R882C, R882S, and R882P [73]. Mutation of the R882 site will alter the activity of DNMT3A, which then affects cell cycle regulation to promote the development of myeloid leukemia. *DNMT3A* mutations occur in about one fifth of acute myeloid leukemia (AML) cases, and these mutations are associated with reduced enzymatic activity and genomic methylation in the leukemia cells [74]. *DNMT3A* mutation often occurs simultaneously with the mutation of other genes, the effects of *DNMT3A* mutation on the methylation of the whole genome are often different. Yan et al. found some differences in DNA methylation pattern in patients with and without the *DNMT3A* R882 mutation [38]. In individuals with the *DNMT3A* R882 mutation, hypomethylation of CpG islands is detected in the *HOXB* locus and genes in the *HOXB* cluster are extensively upregulated at the mRNA level. HOX family proteins play an important role in regulating normal hematopoiesis [75]. In addition, expression of the DNA demethylase IDH1 increased and genome demethylation increased in individuals with the DNMT3A R882 mutation.

DNMT3A was found to be slightly overexpressed in other human cancers [22]. We speculate that this may be related to hypermethylation of TSGs. Deng et al. found that in a mouse model, silencing of *DNMT3A* with RNA interference inhibited melanoma growth and metastasis [76]. Deng’s group evaluated the role of DNA methylation in regulating gene expression. MHC class II transactivator (Ciita), which is essential for T cell-mediated immunity, was upregulated after silencing of *DNMT3A*. Bisulfite sequencing revealed that most CpG sites in the Ciita promoter were significantly demethylated, indicating that the deletion of *DNMT3A* directly mediates the upregulation of Ciita and enhances the immune response. This work also shows that DNMT3A is required for melanoma growth in vivo. Leonard et al. also showed that DNMT3A overexpression in vulvar squamous cell carcinomas increases tumor invasion, and may be useful for predicting the risk of relapse [37].

However, Gao et al. analyzed the role of DNMT3A in lung cancer in genetically engineered mice, and found that deletion of *DNMT3A* significantly promotes oncogenesis [67]. This suggests that in normal cells, the enzyme may act as a TSG to inhibit tumor development by stabilizing DNA methylation patterns, and its deficiency may play a key role in lung cancer malignancies [66,68]. Haney et al. reported that loss of one copy of the *DNMT3A* gene in mice cannot induce the transformation of hematopoietic cells immediately, but can cause chronic lymphocytic leukemia (CLL)-like symptoms in more than half of 16-month-old mice [77]. Global methylation profiling showed that most gene promoters in normal spleen cells are highly methylated and silent, while the loss of one copy of *DNMT3A* induced promoter hypomethylation, leading to overexpression of putative oncogenic drivers. This suggests that *DNMT3A* is a tumor suppressor in CLL.

### 3.3. DNMT3B Aberrations

There are very few reports about *DNMT3B* mutations. Mutations in the human *DNMT3B* gene cause a rare autosomal recessive genetic disease called immunodeficiency-centromeric instability-facial anomalies syndrome 1 (ICF) [78]. Although there is not much evidence for *DNMT3B* mutations in cancer, increased expression of DNMT3B is found in some tumors, which suggests that abnormal expression of DNMT3B may silence TSGs. Robertson et al. reported that DNMT3B was significantly overexpressed in human bladder and pancreatic cancer tissues [22]. It was found that DNMT3B methylates genes that are usually silenced in human colon cancer, this process promotes the formation of intestinal tumors in mouse models [79,80]. Lin et al. investigated the transition of microadenomas (considered to be the earliest stage of intestinal tumor formation) to macroscopic colon tumors in a mouse model. They demonstrated that DNMT3B plays a role in the transitional process [81]. DNMT3B deficiency can apparently reduce the formation of macroscopic adenomas, but it does not reduce microadenoma formation.

Hlady et al. specifically knocked out *DNMT3B* in T cells in a mouse model of MYC-induced lymphomagenesis, thereby increasing cell proliferation and accelerating lymphoma development [82]. Global methylation analysis shows that many gene promoters are potential targets for DNMT3B activity. These promoters are demethylated in DNMT3B-deficient lymphoma, but not demethylated in DNMT3B-deficient precancerous thymocytes, which indicates that DNMT3B maintains cytosine methylation in cancer cells. This work suggests that DNMT3B has an anti-tumor effect in T cells.

## 4. Targeting DNMTs

Since DNMTs are frequently overexpressed in some cancers, DNMTs are excellent targets for treating cancer. As described above, TSG promoters are often hypermethylated in cancer cells. DNMT inhibitors (DNMTis) generally demethylate TSG promoters to facilitate expression of TSGs. Here we introduce different types of DNMTis that inhibit various TSGs and provide a wide range of treatment methods. Depending on the type of cancer, agents that target a single DNMT or multiple DNMTs can be selected. Treatment approaches are mostly divided into two types: the first mainly acts on a single DNMT target, while the second targets DNMTs in combination with other key enzymes, which will greatly improve the efficacy and the likelihood of patient recovery. The two methods are described below in detail.

### 4.1. Targeting a Single DNMT

#### 4.1.1. Targeting of DNMT1 with Decitabine/Shikonin/RG108/DC_05

When a DNMTi is mentioned, the first thought is decitabine (5-aza-2′-deoxycytidine). The cytosine analog 5-aza-2′-deoxycytidine has been approved by the FDA for the treatment of hematological malignancies. When DNA replicates, 5-aza-2′-deoxycytidine is incorporated into the DNA. It can then trap DNMTs and effectively prevent them from methylating the DNA [83]. Hypermethylated TSGs will demethylate and restore the expression of TSGs. The nucleoside drug has a good anti-cancer effect, especially in hematological malignancies, and it also has a good effect in solid tumors. It is reported that aberrant methylation can lead to pancreatic cancer, and overexpression of DNMT1 may be a crucial factor leading to the increased DNA methylation in cancer cells, resulting in abnormal activation of transcription and inhibition of protein degradation. Thus, there is a very urgent need for drugs to treat pancreatic cancer [84]. Epigenetic silencing of *SOCS3* (suppressor of cytokine signaling 3) has a carcinogenic effect and *SOCS3* promoter methylation has been observed in pancreatic cancer. Wang et al. evaluated the methylation status of *SOCS3* gene in 36 patients with pancreatic cancer by MSP analysis. The results showed that *SOCS3* promoter was hypermethylated in one third of the patients, which was closely related to tumor size, tumor differentiation degree and TNM stage. It indicates that SOCS3 methylation is related to the malignant degree of pancreatic cancer [85]. Huang et al. demonstrated that abnormal activation of the IL-6 (interleukin-6)/STAT3 (signal transducer and activator of transcription 3) signaling pathway promotes transcriptional repression of *SOCS3* by increasing DNMT1 activity, which can lead to the growth and metastasis of pancreatic cancer. The use of decitabine to target DNMT1 has delivered a breakthrough in therapy [72]. Decitabine can reverse IL-6 mediated downregulation of SOCS3 by preventing the hypermethylation of the *SOCS3* promoter and increasing its transcriptional activity [33].

Shikonin is a naturally occurring naphthoquinone isolated from the herb purple gromwell (*Lithospermum erythrorhizon*) [86]. It has been reported to show anti-tumor activity in some human cancers [87,88]. Yang et al. noted that shikonin can inhibit thyroid cancer cell growth [89]. Zhang et al. explored the effect of shikonin on DNMT1 in thyroid cancer cells and found that DNMT1 is usually highly expressed and expression of PTEN is low in thyroid cancer. *PTEN* is a classic TSG which negatively regulates the PI3K/AKT signal pathway. It is a lipid phosphatase that dephosphorylates PIP3 (phosphatidylinositol (3,4,5)-trisphosphate) to PIP2 (phosphatidylinositol (4,5)-bisphosphate), This process inhibits AKT activity, thereby downregulating the signaling pathway and inhibiting tumor development [90]. Hypermethylation of the *PTEN* promoter in somatic cells downregulates PTEN expression. The *DNMT1* gene acts upstream of *PTEN* and downregulates *PTEN* via promoter hypermethylation, which promotes the metastasis of human papillary thyroid cancer (TPC-1). Therefore, inhibiting the expression of DNMT1 is a potential therapy for thyroid cancer. It has been experimentally proven that shikonin can inhibit the expression of DNMT1, reduce *PTEN* gene methylation, and increase the expression of PTEN protein, which inhibits the migration of TPC-1 cells. Zhang et al. discovered that shikonin has an inhibitory ability on DNMT1. However, it is not clear how shikonin specifically inhibits DNMT1. It may still be an effective agent to treat thyroid cancer in the future [36].

The non-nucleoside inhibitor RG108 (*N*-phthaloyl-l-tryptophan 1) and selective non-nucleoside DNMT1 inhibitors in the DC_05 series of compounds (DC_501 and DC_517) can also play an anti-cancer role by inducing DNA hypomethylation to restore the expression of TSGs [91,92].

#### 4.1.2. Targeting of DNMT3A with Decitabine/SGI-110/MC3353

Hyperactivity of DNMT3A often occurs in hematological tumors and other tumors. Therefore finding treatments that target DNMT3A has become a research hot spot [93]. Decitabine is one of the most commonly used classical drugs for treating leukemia. It directly inhibits DNA methyltransferase activity to reduce DNA methylation, thereby further inhibiting the proliferation of tumor cells and preventing the occurrence of drug resistance. It is the most powerful known specific DNMT inhibitor. Zhou et al. reported that the gene *BASP1* (brain acid-soluble protein 1) plays a role as a TSG in tumors. Specific downregulation of the *BASP1* gene and methylation of the promoter were observed in AML. The new fusion protein AML1-ETO (A/E) is considered as a possible cause of AML, and the A/E fusion protein can recruit DNMT3A to the *BASP1* promoter region. DNMT3A methylates the *BASP1* promoter sequence and silences *BASP1* expression. Bisulfite sequencing analysis also showed that CpG islands were highly methylated in A/E positive AML cell lines, but not in A/E negative AML cell lines. It suggested that DNA methylation may be involved in transcription silencing of *BASP1* in A/E positive AML. Upregulation of *BASP1* gene expression by decitabine treatment can induce AML cell death [39]. BASP1, as a transcriptional regulator of WT1 (Wilms’ tumor 1) protein, participates in the regulation of many genes related to apoptosis, such as the *BCL*-*2* family, *c*-*Myc* and *p21* [94]. Cashen et al. also reported that a low-dose 5-day decitabine regimen in elderly AML patients has a good early therapeutic effect, less toxicity, and lower 30-day mortality [95].

SGI-110 (guadecitabine) is a second-generation hypomethylating compound, which is a dinucleotide of decitabine and deoxyguanosine linked by a phosphodiester bond. It prolongs the exposure time of the active product decitabine in vivo, thus producing a better effect [96]. It is also commonly used to treat AML diseases. As well as demethylating CpG-island promoters, it can also enhance the sensitivity of tumor cells to some anticancer drugs [97]. In hepatocarcinoma, Jueliger et al. used Luminometric Methylation Assay to analyze global DNA methylation, and found that the whole genome methylation levels of HepG2 and Huh-7 cells were similar. The promoters of the tumor suppressor genes *MTS1* (multiple tumor suppressor 1 or p16), *DLEC1* (Deleted in lung and oesophageal cancer 1) and *RUNX3* (RUNX family transcription factor 3) are hypermethylated. Jueliger et al. discovered that SGI-110 demethylates the promoters of the tumor suppressor genes *MTS1*, *DLEC1* and *RUNX3* and achieves an anticancer effect [98]. Similarly, a new quinoline-based molecule, MC3353, can also inhibit the activity of DNMT1 and DNMT3A to reverse hypermethylation caused by cancer [99].

#### 4.1.3. Targeting of DNMT3B with Nanaomycin A

There are many non-selective inhibitors that can inhibit DNMT3B along with other DNMTs, but here we introduce a rare selective DNMT3B inhibitor. Nanaomycin A is the first selective DNMT3B inhibitor which can induce genomic demethylation. It interacts with amino acid residues in DNMT3B which are involved in methylation, so that DNMT3B cannot participate in normal DNA methylation [100]. Nanaomycin A demethylated the DNA and reactivated the transcription and expression of the gene *RASSF1A* (ras association domain family 1 isoform A). *RASSF1A* is a TSG frequently silenced by promoter hypermethylation in cancer [101]. Kuck et al. noted that in lung cancer cells, the promoter region of RASSF1A was sequenced by bisulfite sequence, which confirmed that RASSF1A promoter was highly methylated in A549 cells [40]. Hepatocellular carcinoma (HCC) is a cancer with a high mortality rate worldwide, and patients are prone to drug resistance and poor prognosis in treatment [102]. This is mainly because increased expression of DNMT3B mediates increased expression of the *OCT4* gene (octamer-binding transcription factor 4) through the IL-6/STAT3 pathway in HCC cells. Lai et al. found that the activation of p-STAT3 increased the resistance of HCC cells to sorafenib and explored whether nanaomycin A also achieves a good therapeutic effect in HCC. They demonstrated that nanaomycin A targeted DNMT3B to inhibit OCT4 expression and increase the sensitivity of drug-resistant cells. The *OCT4* promoter is typically hypermethylated in normal liver cells. However, the methylation status of oncogene *OCT4* during carcinogenesis still remains unclear. Perhaps there are other mediators between DNMT3B and OCT4 [41].

### 4.2. Combination Targeting Approaches

#### 4.2.1. Combination of Decitabine and HDACi

The decline in the efficacy of decitabine alone has led to the emergence of combination therapies. The effect of decitabine combined with a histone deacetylase inhibitor (HDACi) has been gradually verified in different tumors, such as AML [103]. As DNMTis are common drugs for the treatment of hematological malignancies, we will not introduce more leukemia-related applications here. Instead, we want to present more extensive applications, so we will now focus on solid tumors. Lung cancer is one of the most frequently occurring malignancies in the world, and its incidence rate and mortality rate are the highest of all cancers [104]. In general, drugs are the first choice for treating lung cancer. However, in cases where the expression of *DNMT* genes is altered, the effect of drug treatment is reduced and the condition becomes worse.

As described above, evidence suggests that overexpression of DNMTs induces DNA methylation and silencing of TSGs, leading to malignant lung cancer. Forced expression of DNMT1 or DNMT3A increases DNA methylation, while elimination of DNMT expression by small molecule inhibitors reduces DNA methylation of the entire genome and specific genes. More importantly, it restores the expression of TSGs. Their silencing predicts poor results by promoter hypermethylation. In order to change this hypermethylation state, Belinsky et al. proposed that combining decitabine with an HDACi (sodium phenylbutyrate) would restore the normal methylation state in murine lung cancer, including the re-expression of TSGs that are densely hypermethylated and transcriptionally silenced [61]. Similarly, Vendetti et al. proposed epigenetic treatments that target DNMTs and HDACs in non-small cell lung cancer [105].

Among malignant tumors of the female genital tract, ovarian cancer is the third in terms of incidence and mortality. Thus, it is essential to understand the molecular mechanisms underlying ovarian cancer and find effective drugs for the treatment of this highly metastatic and deadly disease [106]. Zhao et al. found that the increased expression of DNMT3B in ovarian cancer cells increased the methylation level of the promoter regions of anti-oncogenes such as *hMLH1* (mismatch repair gene human mutL homolog-1), *p16* and *p53*. These methylation changes decreased the expression of the *hMLH1*, *p16* and *p53* genes and promoted tumor development [42,107]. As mentioned above, decitabine is a specific DNMT inhibitor, which can partially reverse DNA methylation. Steele et al. noted that the effect of decitabine on demethylation is limited by the dose and the eventual remethylation of the gene. They also noted that histone acetylation is related to chromatin opening, which helps to promote gene transcription and is closely involved in the proliferation, differentiation and progress of tumor cells. Therefore, they used decitabine with a clinically effective inhibitor of histone deacetylase activity (belinostat), and found that this combination restored the expression of epigenetically silenced genes [108].

#### 4.2.2. Combination of Decitabine and GSK126

EZH2 is the catalytic subunit of the polycomb repressive complex 2 (PRC2), which regulates histone methylation (H3K27me3) [109]. EZH2 is often dysregulated in melanoma, the deadliest skin cancer, and it has been shown to interact with DNMT and cause hypermethylation of TSG promoters, resulting in silencing of TSGs [110]. Tiffen et al. found DNA methylation and H3K27me3 on some EZH2 target genes. Moreover, treatment of melanoma cell lines with GSK126 alone did not restore the expression of EZH2 target genes, and there was no significant change in DNA methylation in TSG promoter regions, which proves that DNA hypermethylation inhibits the expression of EZH2 target genes in melanomas. Tiffen et al. speculated that the combination of decitabine and GSK126 would result in a more effective upregulation of EZH2 target genes compared to either inhibitor alone. After the combined treatment, it was found that two EZH2 target genes were upregulated, namely the tumor suppressor gene *RASSF5* (ras association domain family member 5) and *ITGB2* (integrin subunit Beta 2), which is associated with anti-tumor immunity [111,112]. The results showed that EZH2-driven hypermethylation silenced these two target genes. This may be due to the direct interaction between DNMTs and EZH2 protein domains, or to the recruitment of EZH2-containing PRC2 complex to unmethylated CpG islands. The combined use of a DNMTi and an EZH2 inhibitor (EZH2i) may lead to expression of tumor suppressor genes and increased immune responses to effectively treat melanoma [49].

#### 4.2.3. Combination of AZA and RPM

DNMT-mediated abnormal DNA methylation is a common epigenetic change in the early stages of tumorigenesis and is closely related to the development of colorectal cancer (CRC), the third most frequently diagnosed malignancy worldwide. Most colorectal cancers have no obvious symptoms in the early stage and are difficult to treat in the later stage [113]. Rodriguez et al. found that DNMT suppression activates gene promoter regions (especially TSG promoters) after demethylation, thereby inhibiting cancer growth [114].

Zhang et al. noted that mammalian target of rapamycin (mTOR) plays a critical role in cell growth and homeostasis, and it is often altered in cancers. Based on these reasons, mTOR is currently being studied as a potential target for anti-cancer therapy. Rapamycin (RPM) is an mTOR inhibitor (mTORi) and has been proved to have anti-tumor activity. Zhang et al. investigated whether a combination of RPM and other inhibitors has additive effects on tumor growth. They found that combining RPM with 5-aza-deoxycytidine (AZA) effectively eliminated the downstream effects of mTOR activation in colorectal cancer cell lines. The mechanism may relate to the regulation of tumor suppressor factors, such as PTEN, which play a role upstream of the PI3K/AKT/mTOR signaling pathway, leading to significant apoptosis as well as cell cycle arrest. In addition, in a mouse CRC model, the combination of AZA and RPM reduced the incidence of CRC and the tumor volume, and decreased the activity of the mTOR signaling pathway. The results showed that a DNMTi combined with an mTORi had inhibitory effects on colorectal cancer cells in vivo and in vitro. Such combinations may help to optimize the overall benefits associated with molecularly targeted anti-cancer drugs [115].

#### 4.2.4. Dual Inhibitors

Combining the effects of two inhibitors in the same compound will achieve better efficacy. C02S is such a drug and may be used to treat breast cancer, which is the second most common cause of cancer-related deaths in women [116]. Most breast cancers have a poor prognosis, are prone to drug resistance, and lack targeted agents [117]. Some breast cancer cells exhibit a genomic hypermethylation phenotype, which is caused by the abnormal activity of DNMTs. High expression of DNMT3B protein is related to the overall increase in DNMT activity, thus inducing highly methylation of multiple genes (*CDH1*, *CEACAM6*, *CST6*, *ESR1*, *LCN2* and *SCNN1A*) [43]. It has also been reported that overexpression of DNMT3B increases the recurrence of breast cancer in patients treated with tamoxifen [118]. Yuan et al. noted that DNMTs and HDACs respectively catalyze the methylation of CpG islands in DNA and the deacetylation of histones and other substrate proteins. Abnormalities in these enzymes are related to the reduced expression of TSGs (*p16*, *p21* and *TIMP3*), and their interaction reinforces the silencing effect of TSGs. Yuan et al. found that the compound C02S, which acts as a dual DNMTi and HDACi, was effective in breast cancer cells and downregulated multiple cancer-related processes. These results suggest that C02S may be a means of treating breast cancer in the future [116].

In addition, CM-272 is a potent dual inhibitor of G9a (a type of histone methyltransferase) and DNMTs for the treatment of hematological malignancies [119]. G9a is responsible for methylating lysine 9 of histone 3 (H3K9), and overexpression of G9a leads to increased tumor progression in some tumors [120,121]. It is involved in the transcriptional repression of the tumor suppressor gene *PTEN*, and upregulation of the *G9a* gene and downregulation of the *PTEN* gene can predict poor overall survival [122]. Decreasing the methylation level of DNA and H3K9 can cause reactivation of PTEN and inhibit cancer cell proliferation. San et al. synthesized a compound, CM-272, to simultaneously target G9a and DNMTs as an improved approach for cancer treatment. They demonstrated that CM-272 inhibited the proliferation of different hematological cancer cells and promoted cancer cell apoptosis. Dual inhibition of G9a/DNMTs represents a novel method for safely and effectively targeting cancer [119].

## 5. Conclusions and Perspectives

It is known that DNA methyltransferases play an essential role in life and participate in the development of many cancers. This review has systematically introduced the characteristics of DNMTs, the DNMT3A paradox and the different roles of DNMTs in cancers, and the strategies that are available for targeting DNMTs in cancer therapy.

We propose that the DNMT3A paradox may result from the different characteristics of DNMT3A in different processes. On the one hand, DNMT3A plays a critical role in promoting tumorigenesis by hypermethylation of certain regions, such as the promoters of TSGs. Decreased activity of DNMT3A causes DNA demethylation, and TSGs may be re-expressed to suppress tumors via an increase of microRNAs [64,123]. On the other hand, DNMT3A guards the stability of the genome by maintaining global genomic hypermethylation. In this respect, it is equivalent to a tumor suppressor gene. When the *DNMT3A* gene is deleted by experimental means, the methylation level decreases and the CpG sites originally modified by methylation are hypomethylated. Thus, the chromatin structure opens up, which makes the genome unstable and susceptible to alteration. The degree of malignancy will increase [66,67,68]. In addition, we suspect that the paradox is maybe due to various types of lung cancer cells with different inherent mutations, such as *EGFR* mutation, *KRAS* mutation, *TP53* mutation, etc [124]. It is worth considering whether *DNMT* genes and these genes will produce synergistic or antagonistic effects, which will lead to distinct functions of DNMT.

**Figure 4:**
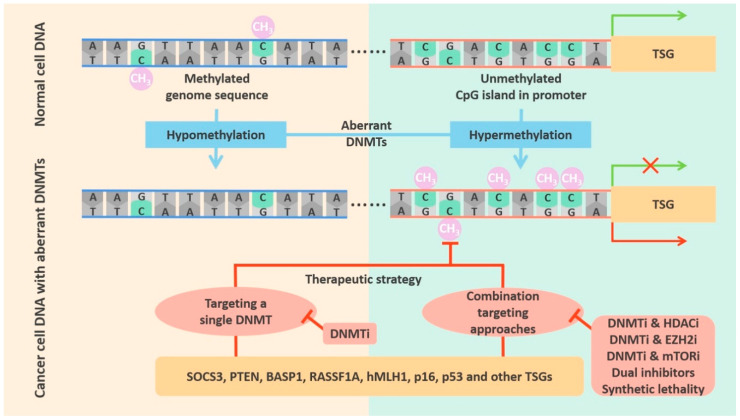
Strategies for targeting DNMTs in cancers. In normal cells, the genome sequence is usually methylated, and the promoter regions of TSGs are unmethylated. In tumor cells, DNMT aberrations cause hypomethylation of the genome sequence and hypermethylation of TSG promoters. There are two therapeutic strategies: one is to use a DNMTi that targets a single DNMT, and the other is to use a combinatorial approach, such as DNMTi + HDACi, DNMTi + EZH2i, DNMTi + mTORi, dual inhibitors, or synthetic lethality.

Since DNMT overexpression often occurs in cancer, many researchers have tried to use drugs to specifically target DNMTs for therapeutic purposes. The whole-genome hypomethylation in cancer cells can be restored to normal levels by targeting DNMTs in a variety of tumors. Targeting of DNMTs can also reverse the hypermethylation of TSG promoter regions which is seen in many cancers. This targeted therapy is powerful and widely applicable to different cancers. In addition to targeting DNMTs alone, it is possible to use combined treatments to improve the effect (see Figure 4).

However, there are some potential drawbacks of using DNMTi. On the one hand, clinical trials and clinically approved DNMTis are mostly applicable to hematological tumors. In addition to decitabine, which is often mentioned above, there is guadecitabine for the treatment of relapsed/refractory AML [125], and the combination of DNMTi CC-486 and HDACi romidepsin for the treatment of advanced solid tumors [126]. These drugs are also very promising in clinical applications. In our review, for some cancers, targeted DNMT agents may not be the most suitable and some drugs can inhibit multiple DNMT members simultaneously. However, we want to provide a novel idea to treat multiple cancers in order to increase the diversity of therapies. On the other hand, this method only targets the particular tumors of overexpressed DNMTs and can prevent hypermethylation of TSGs promoter. It is not suitable to use DNMT inhibitors to treat the cancers caused by decreased DNMTs activity, and the therapies for this type of cancers need to be further explored.

The previous results from our group have demonstrated that *DNMT3A* acts as a tumor suppressor gene in lung cancer. We knocked out *DNMT3A* using the CRISPR-Cas9 system and found that it participates in cell proliferation, migration and invasion. However, the high mutation rate of *DNMT3A* always results in loss of DNMT3A function, and it is difficult to target loss-of-function mutations. Furthermore, although DNMT inhibitors have some effect in solid tumors, they are not as effective as in hematological malignancies [61,127], and therefore we still do not have an effective cure for lung cancer. We are looking forward to identifying some effective molecular targets and extending them to the therapy of other types of cancer. We speculate that it will be possible to develop new therapeutic approaches, such as synthetic lethality [128]. We can use the CRISPR-Cas9 system, shRNA technology or inhibitor libraries for high-throughput screening [129,130], and identifies multiple epigenetic-related synthetic lethal pairs [131,132,133]. Cancer cells with “undruggable” mutant genes can be selectively killed by specifically targeting the function of a synthetic lethal partner [134]. Perhaps we can find new DNMT-targeted tumor treatments through a synthetic lethality strategy (see Figure 4).

In summary, this review presents the biological characteristics of DNMTs, their alterations in different malignancies, and therapies that target them. In addition, we have proposed more ideas for targeting DNMTs, which may provide the foundation for future cancer treatments.

## Figures and Tables

**Table 1:** Expression levels of DNMTs in normal tissues and deregulation of DNMTs in cancers.

DNMT	High Level in Normal Tissues	Low Level in Normal Tissues	Cancers with Deregulated DNMTs	Nature of Deregulation in Cancers	Result
DNMT1	Fetal tissues, heart, kidney, placenta, peripheral blood mononuclear cells	Spleen, lung, brain, small intestine, colon, liver, skeletal muscle	Pituitary adenoma	Overexpression	Silenced *RASSF1A*, *CDH13*, *CDH1*, and *p16* [31]
			Colorectal cancer	Mutation	Alteration of global DNA methylation status [32]
			Pancreatic cancer	Overexpression	Transcriptional repression of *SOCS3* in abnormal activation of the IL-6/STAT3 pathway [33]
			Gastric cancer	Overexpression	Hypermethylated CpG islands of the *p16*, *hMLH1*, *E-cadherin* and *THBS-1* [34]
			Lung cancer	Overexpression	Hypermethylation of *p16*, *RARβ*, *FHIT*, *RASSF1A* and *hRAB37* with aberrant p53/Sp1 axis [35]
			Thyroid cancer	Overexpression	Downregulation of *PTEN* by DNA methylation in PI3K/AKT signal pathway [36]
DNMT3A	Fetal tissues, skeletal muscle, heart, peripheral blood mononuclear cells, kidney	Placenta, brain, liver, colon, spleen, small intestine, lung	Vulvar squamous cell carcinoma	Overexpression	The absence of p16 expression by promoter hypermethylation [37]
			Pituitary adenoma	Overexpression	Silenced *RASSF1A*, *CDH13*, *CDH1*, and *p16* [31]
			Acute myeloid leukemia	Mutation	DNA hypomethylation by *DNMT3A R882* mutation [38]
				Overexpression	Transcription silencing of *BASP1* in A/E positive AML [39]
DNMT3B	Fetal liver, heart, kidney, placenta	Spleen, colon, brain, liver, small intestine, lung, peripheral blood mononuclear cells, skeletal muscle	Lung cancer	Overexpression	Highly methylation of *RASSF1A* promoter [40]
			Hepatocellular carcinoma	Overexpression	Increased expression of the oncogene *OCT4* through the IL-6/STAT3 pathway [41]
			Ovarian cancer	Overexpression	Hypermethylation of *hMLH1*, *p16* and *p53* [42]
			Breast cancer	Overexpression	Highly methylation of multiple genes (*CDH1*, *CEACAM6*, *CST6*, *ESR1*, *LCN2* and *SCNN1A*) [43]
